# Editorial: Peptides Targeting Protein-Protein Interactions: Methods and Applications

**DOI:** 10.3389/fmolb.2021.780106

**Published:** 2021-10-14

**Authors:** Laura Belvisi, Luca Domenico D’Andrea, María Angeles Jiménez

**Affiliations:** ^1^ Dipartimento di Chimica, Università Degli Studi di Milano, Milano, Italy; ^2^ Istituto di Scienze e Tecnologie Chimiche “G. Natta”, Consiglio Nazionale Delle Ricerche, Milano, Italy; ^3^ Instituto de Química Física Rocasolano (IQFR), CSIC, Madrid, Spain

**Keywords:** peptide, protein-protein interaction, computational design, bioactive peptide, molecular recognition

## Introduction

Proteins perform a myriad of crucial roles in biological systems that depend mainly on the interaction with one or more proteins. Many physiological and pathological conditions are regulated by recognition and association among proteins. Therefore, the development of molecules able to modulate these protein-protein interactions (PPIs) open the way towards novel drugs, diagnostics and molecular tools for chemical biology and therapeutic applications. Peptides represent a class of compounds well suited for protein recognition and, of course, to modulate protein-protein interactions. More, considering the pharmaceutical field peptides are the best candidate to fill the gap between small molecule drugs and protein therapeutics.

This Research Topic aims to provide an overview of the current state and recent advances of the field. To that end, it includes both review and original research contributions that highlight promising and compelling computational tools developed to design bioactive peptides/peptidomimetics as well as successful examples of peptides, which targeting a protein-protein recognition site, are able to modulate a biological response or bind to a specific protein.

## Computational Approaches to Design Protein-Targeting Peptides

Peptides targeting PPIs have been found by serendipity, screening libraries or designed by structure-based computational methods. In particular, the *in silico* approaches are gained much popularity as the methodology is coming robust. In this Topic Hashemi et al. review the recent advances in the field of in silico approaches for the design of interfering peptides against PPIs, covering many aspects and stages of the peptide design process. The review by Pérez et al. summarizes well established computational tools used to study protein/peptide interactions and provide examples of their applications in conjunction with other techniques to a few cases in drug discovery and tissue engineering. Hassankalhori et al. describes novel frontiers in computational studies applying their Supervised Molecular Dynamics protocol to explore the molecular recognition and the binding mode of macrocyclic peptides to thrombin.

## Applications of Protein-Targeting Peptides

Modulation of PPIs constitutes a promising, but challenging, way to find novel therapeutic agents to fight numerous pathologies. The fact that PPIs exhibit large surface contact areas, but lack deep binding cavities, make them difficult to target for with small molecules. Because of their physical properties, peptides are, instead, more suitable to target PPIs. The contributions included in this Topic provide a view of the advances in finding peptide modulators. The review by Cabri et al. provides us quite a thorough overview of the peptides that are currently in different phases of clinical development. These peptides are directed to diverse pathologies, i.e. cancer and related diseases, inflammation and autoimmune disorders, genetic and hormonal diseases, diabetes, obesity, cardiovascular, and neurodegenerative diseases, as well as bacterial and viral infections. Pluda et al. reviews linear and cyclic peptide-based inhibitors of two metalloproteinases, whose dysregulation leads to several pathologies (cancer, inflammatory, neurodegenerative, and cardiovascular diseases). The strategies to find these peptides include traditional medicinal chemistry, rational design, and combinatorial peptide display technologies. The review by Yang et al. focuses on the interaction between APC and ASEF, two proteins involved in metastatic colorectal cancer, and their structure-based designed peptide inhibitors. Alexa et al. describe their experimental results on finding peptides inhibiting mitogen-activated protein kinases (MAPK), involved in the regulation of many cellular functions such as cell division, differentiation and apoptosis. A 15-mer peptide derived from the RHDF1 protein, which is able to bind MAPK, is taken as starting hit and modified to optimize cell-penetrating capacity and decrease its length without affecting its binding affinity. Orea-Ordóñez et al. proposes to disrupt ribosome assembly as a novel approach for cancer therapy. Based on the crystal structure of the complex between the Erb1 and Ytm1 ribosome assembly factors, a series of peptides potentially able to interfere with that interaction are designed and assayed *in vitro*. Singh et al. focus on the interaction between a voltage-gated Na^+^ channel (NAV1.6) and the fibroblast growth factor 14 (FGF14), which participates in the regulation of neuron excitability in clinically relevant regions of the central nervous system. They found that two 4-mer FGF14-derived peptides inhibit the formation of the FGF14/NAV1.6 complex.

## Future Perspectives

On the whole, the review and original research contributions gathered in this Research Topic provide an excellent view of the current state of the field of peptides targeting protein-protein interactions. Based on them, we can envisage a growing and successful future of the field. The modulation of PPIs with peptides has turned out a valuable approach for a number of different pathologies with many peptides already in clinical development, however the area is expected to broaden and improve applications in the near future as a huge number of PPIs are still unexplored or unknown. In this framework the availability of powerful and innovative methodologies, both experimental and computational, can support the progresses in the challenging effort of discovering novel peptide as PPI modulators endowed of optimal properties.

